# A systematic search and review focusing on the influence of health-tracking technologies on eating habits and attitudes

**DOI:** 10.1177/13591053251351222

**Published:** 2025-07-16

**Authors:** Tamara Wallace, Christian Koebbel, Jennifer Heath

**Affiliations:** 1 Oxford Health NHS Foundation Trust, UK; 2 East London NHS Foundation Trust, UK; 3 University of Hertfordshire, UK

**Keywords:** disordered eating, eating behaviour, health-tracking technology, relationship with food, self-monitoring

## Abstract

A systematic search and review of studies of health-tracking technologies that included diet-related outcomes, in adult non-clinical populations, was conducted to produce an evidence-based response to the question, ‘What does the literature tell us about experiences of health-tracking technologies in relation to relationship with food and eating in non-clinical populations?’. Fifteen studies were included in the review and five recurring concepts were identified in relation to the experiences of health-tracking technology users; these were: 1-motivators and barriers to use, 2-process of logging food, 3-reification of data, 4-disordered eating and compulsive exercise and 5-goal achievement and positive self-appraisal. Self-monitoring of food intake impacts eating behaviour and attitudes towards eating, in terms of adoption of short-term healthy habits and the development of disordered eating habits. Although health-tracking apps/devices play a role in improving public health, there are populations for which dietary self-monitoring is associated with disordered eating and negative mental health outcomes.

## Introduction

‘Self-tracking’ and ‘quantified self’ are frequently used terms describing use of health-tracking technologies. Increasingly, these technologies are used by lay people as voluntary self-tracking strategies that contribute to perceptions of health, embodiment, and identity, and can be viewed as part of managing and improving one’s life (Lupton, 2013).

Literature refers to health-tracking technologies in multiple ways, but for the purposes of this systematic literature review, ‘health-tracking technologies’ encompasses ‘fitness-tracking’ or ‘activity-tracking’ technologies designed to record daily physical activity, and ‘calorie-tracking’ or ‘food-tracking’ technologies to record daily dietary consumption. Use of such trackers is grounded in research suggesting that technology increases self-awareness of health-related behaviours, which motivates behavioural change (Chen et al., 2018).

Health-tracking technologies are often in the form of wearables (e.g. FitBit) or mobile apps (e.g. MyFitnessPal). The FitBit is an activity monitor that has demonstrated high validity and reliability for energy expenditure estimates (Lee et al., 2014) and the companion app allows users to log food intake and access visual feedback. The MyFitnessPal app has a reported 180 million users worldwide (MyFitnessPal, 2019) and allows users to create a diet plan by entering certain characteristics and goals. Users enter their consumed food and can input exercise manually or by synchronising other apps, such as the FitBit. As with many health-tracking technologies, both also offer connection with other users through social media.

When considering the impact of health-tracking technologies on users’ relationship with food, it is notable that such technologies are commonly used in weight loss interventions. A systematic review of research comparing the effectiveness of weight loss practices with and without mobile apps found broad support for their use, suggesting participants aided by an app lose more weight because of better nutrition decisions and increased activity (Lyzwinski, 2014). Self-tracking can offer a memory aid for daily consumption or facilitate consideration of whether certain foods fit into their diet plan. Others may log planned meals, avoiding spontaneous decisions about food. Furthermore, the process of logging every mouthful may prompt momentary evaluation of the food item as opposed to automatic consumption (Wang et al., 2021). It appears then that health-tracking technologies can alter an individual’s relationship with and behaviour around food, at least during pursuit of short-term goals.

Although there are indications that dietary self-monitoring can positively impact food choices and behaviour change (El Khoury et al., 2019), health-tracking technologies are not void of concerns. A significant issue with activity trackers in particular is that they do not build in rest days from monitoring. The focus on continuous tracking can encourage prioritising numbers rather than feedback from the body. This can be unhelpful for individuals vulnerable to disordered eating, mirroring the practice of ignoring signals from the body, such as hunger or pain, which is typical in eating disorder presentations (Brown et al., 2020).

Most existing health-tracking technologies were not designed for individuals with a history of eating disorder and have not been robustly evaluated in these populations (Levinson et al., 2017). Given that individuals with current or historical eating disorders may be drawn to perfectionistic habits around calorie counting and numbers, it is important to understand the impact of technology. However, the literature offers inconsistent conclusions. It has been suggested that MyFitnessPal should not be used by individuals with eating disorders as it can lead to over-evaluation of weight and shape and restrictive, unbalanced diets (Evans, 2017). In two cross-sectional studies, almost three quarters of female participants with eating disorders viewed MyFitnessPal as, at a minimum, having somewhat contributed to their eating disorder (Levinson et al., 2017). Conversely, an experimental study found no evidence for a causal effect of using MyFitnessPal on eating disorder symptomology (Jospe et al., 2017), and apps can be used to promote recovery via tracking macronutrient intake.

Overall, literature suggests that, while some users of health-tracking technologies may experience thought cycles that lead to new insights (i.e. self-reflection), others may also experience negative thought cycles associated with disordered eating, guilt, and shame (Eikey et al., 2021). Users who experience negative emotions and cognitions in response to their self-monitoring may abandon the technology to avoid such experiences or become stuck in disordered habits.

Given the rise in wearable health-tracking technology, this review of empirical literature aims to systematically gather, organise and evaluate existing studies that have reported on the impact of food- and activity-tracking technologies on eating behaviour. The approach of a systematic search and review combines the strengths of a critical review with a comprehensive and transparent search process (Grant and Booth, 2009), enabling the authors to present a ‘best evidence synthesis’ regarding the impact of food- and activity-tracking technologies on eating behaviour, addressing the question: What does the literature tell us about experiences of health-tracking technologies in relation to relationship with food and eating in non-clinical populations? Better understanding of the potential intended and unintended impact of health-tracking technology on eating behaviour in non-clinical populations can inform assessment, outcome monitoring, and development of support around the use of such tracking technologies within healthcare sectors.

## Method

Ethical statement: As this is a review, ethical approval is not required. There are no human participants in this article and informed consent is not required.

Systematic searches were conducted in January 2022 and repeated in January 2023 using the bibliographic databases: Scopus, Cinahl Plus, and PubMed, chosen as they incorporated literature from various disciplines relevant to healthcare: medicine, nursing and applied social sciences. Considering the rapid development of technology and continuously updating smartphones, literature from over a decade ago was deemed less relevant, although quantitative, qualitative and mixed method studies were included in order to provide knowledge produced from a wide range of study designs (Grant and Booth, 2009). Table 1 provides a summary of the study inclusion and exclusion criteria. Several pilot searches identified key search terms that informed the final search strategy (Table 2). Author TW screened all records and extracted data from each record. The review included health-tracking technologies collecting continuous data and requiring user input, and studies had to report outcomes specific to eating habits or attitudes. Not included were studies using diet-related text-message reminders and studies that involved clinical interventions monitored by health-tracking technologies (e.g. weight loss intervention).

**Table 1.** Inclusion and exclusion criteria.

Inclusion criteria	Exclusion criteria
Published since 2011 Focused on the experience of health-tracking technology users Device or mobile application that collects continuous data or requires user input Device or mobile application that collects data on food consumption Reporting original peer-reviewed research Qualitative, quantitative, or mixed methods Adult or older adult population English language paper	Published before 2011 Conceptual or theory Clinical population, e.g. physical health conditions, diagnosis of eating disorder or obesity Self-monitoring as part of medical intervention e.g., to monitor heart arrythmia, weight loss Paediatric population

**Table 2.** Search strategy.

Concept 1: Terms relating to health-tracking technologies		Concept 2: Terms relating to eating behaviour		Concept 3: Terms relating to experiences
Fitbit OR ‘apple watch’ OR myfitnesspal OR ‘my fitness pal’ OR ‘wearable tech*’ OR ‘wellness device’ OR Garmin OR ‘wellness track*’ OR ‘activity track*’ OR ‘wellbeing monitor’ OR ‘fitness monitor’ OR ‘fitness track*’ OR ‘health track*’ OR ‘digital health’	AND	Food OR eat OR eating OR diet* OR ‘eating disorder’ OR ‘disordered eating’ OR restrict* OR ‘calorie counting’ OR meal OR ‘meal plan*’ OR consum*	AND	experience OR narrative OR impact OR psychosocial OR attitude OR opinion OR perce* OR belie* OR feel* OR know* or understand*

Filter for: participants 18+, human participants, English language, published in or after January 2011.

### Systematic search process

**Figure 1.**
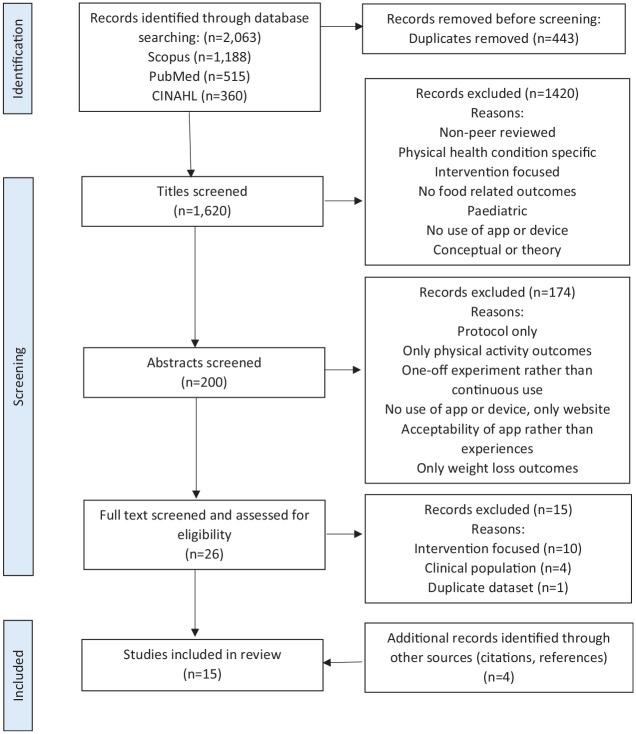
PRISMA 2020 flow diagram of study selection process.

Figure 1 presents a flow chart of the search results and PRISMA screening process (Page et al., 2021). An exhaustive and comprehensive search was conducted to identify, appraise, and synthesise the existing research evidence in line with the review process described by Grant and Booth (2009) and reported using the PRISMA framework.

The initial search identified 1647 articles, of which 389 duplicates were removed. Screening titles and abstracts against inclusion criteria removed a further 1234 articles with 24 remaining for full-text screening. Of these, nine met criteria for inclusion in the review. The reference lists of selected articles were hand-searched to check for others that met inclusion criteria; an additional four articles were identified. When the search was repeated in January 2023, 416 new papers were identified and screened, two of which met inclusion criteria. Therefore, 15 articles are included in the current review. This sample features 10 studies using quantitative methods (Bracken and Waite, 2020; Gittus et al., 2020; Hahn et al., 2021a, 2021c, 2022; Linardon and Messer, 2019; Plateau et al., 2018; Simpson and Mazzeo, 2017; Slazus et al., 2022; Sarcona et al., 2017), four studies using qualitative methods (Didžiokaitė et al., 2018; Hahn et al., 2021b; Lieffers et al., 2018; Régnier and Chauvel, 2018) and one study using mixed methods (Honary et al., 2019).

Quality appraisal of included studies employed four method-specific quality appraisal tools, which provides a useful informal evaluative component in the review (Grant and Booth, 2009). Qualitative studies were quality appraised using the ‘Big-Tent’ Criteria for Excellent Qualitative Research (Tracy, 2010); due to the range of approaches used (e.g. semi-structured individual interviews and workshops), it was important to use an appraisal framework that conceptualises different qualitative methodological paradigms. Of the eight quantitative studies, the two RCTs were appraised using the Critical Appraisal Skills Programme (CASP) Checklist for Randomised Controlled Trials (Critical Appraisal Skills Programme (CASP), 2020), and the six cross-sectional survey studies were appraised using the Appraisal tool for Cross-Sectional Studies (AXIS; Downes et al., 2016). The mixed methods study was appraised using the Mixed-Methods Appraisal Tool (MMAT; Hong et al., 2018). The MMAT criteria for qualitative and quantitative studies are less comprehensive than other method specific criteria, however the MMAT was used to evaluate both arms of the study and combine these in one overall evaluation.

### Included study aims

Research aims of included studies included exploring experiences of health-tracking technologies in university students (Hahn et al., 2021a, 2021b, 2021c, 2022; Sarcona et al., 2017; Simpson and Mazzeo, 2017; Slazus et al., 2022), investigating experiences, goal achievement or eating pathology in adults who were existing health-tracking technology users (Bracken and Waite, 2020; Didžiokaitė et al., 2018; Honary et al., 2019; Lieffers et al., 2018; Linardon and Messer, 2019; Plateau et al., 2018), examining experiences of Fitbit self-tracking (Gittus et al., 2020), and determining conditions under which adults were more likely to use health-tracking apps (Régnier and Chauvel, 2018).

### Synthesis strategy

The review synthesises the 15 articles’ findings using guidance from Siddaway et al. (2019). Author TW led on the synthesis process, reviewed by authors JH and CK. As the synthesis used extracted information related to the specific research question, study design or appraisal of study quality did not lead to differential weighting of findings. Details pertaining to research design, setting, and participants have been included in the narrative.

Following familiarisation with the papers, prominent or recurring observations were noted. Through discussion with authors JH and CW, potential themes were generated and checked against the evidence presented in the reviewed articles. Following further review of themes, central and recurring concepts were identified and grouped, with theme names chosen, to represent the specific experiences of health-tracking technology users participating in the selected studies. The themes provide a comprehensive narrative of what is known from the reviewed research evidence, to make recommendations for practice, and to address limitations of the evidence and also the review process itself (Grant and Booth, 2009).

### Synthesis of findings

The following themes, produced as a result of the synthesis, structure the narrative that follows: (1) Motivators and Barriers to Use, (2) Process of Logging Food, (3) Reification of Data, (4) Disordered Eating and Compulsive Exercise and (5) Goal Achievement and Positive Self-Appraisal. No papers were considered poor quality during quality appraisals such that their findings should be considered with caution.

### Motivators and barriers to use

Where participants were already health-tracking technology users, references were made to participants’ reasons for initiating use. Didžiokaitė et al. (2018) found the majority of their sample of 31 adults reported using MyFitnessPal for dieting. Honary et al. (2019) found the most common reasons for use in their sample of 106 survey participants were calorie-tracking (31%), weight loss (20%), and exercise (21%). Lieffers et al.’s (2018) sample of 24 participants were mostly interested in weight loss or maintenance. Plateau et al.’s (2018) sample of 352 university students reported using activity-tracking devices for either health and wellbeing, weight and shape reasons, or sporting goals. Of those using food-tracking devices, 30% did so for health and 70% to manage weight and shape. Slazus et al. (2022) found most of their 991 survey participants wanted to improve their diet to be healthier (90%) and to improve their weight (74%). Overall, it appears that weight loss or appearance concerns were the most popular motivators to initiate use of health-tracking technologies, followed by exercise and sport performance goals. In this way, individual goals may influence the type of app chosen (i.e. activity-tracking or food-tracking; Plateau et al., 2018). Sex differences were also identified in two papers; a higher percentage of women reportedly used both activity-tracking and food-tracking apps compared to men (Hahn et al., 2022), and men appeared to more frequently pursue weight gain or maintenance, whereas women pursued weight loss (Slazus et al., 2022).

Régnier and Chauvel (2018) investigated motivators for use via exploration of the social, economic, and cultural conditions in which adults in France were more likely to use health-tracking technologies, identifying three clusters of users. The first ‘resistant’ cluster encompassed individuals who monitored dietary intake or physical activity, but recorded very little. Most of these users were considered to be living in deprived areas and temporary app use was reported to cease because of disinterest or reaching their weight goal. The second ‘self-improvement’ cluster consisted largely of sport app and MyFitnessPal users who appeared to value quantifying themselves and measuring progress. The third ‘sharing’ cluster encompassed users who actively engaged with social media, published their data and tended to be professionals in their 40s. Régnier and Chauvel (2018) highlighted that individuals from affluent social milieus were most likely to use apps with a preventative approach to healthy living, whereas individuals from lower milieus often reluctantly used apps for weight loss. It is clear that financial circumstances can influence the technology available to the user, be it costly sport performance devices or free food-tracking apps, as well as potentially the quality of food options available.

Several articles identified that use of health-tracking technologies was often temporary, as opposed to lifelong self-tracking. Didžiokaitė et al. (2018) reported that their interviewees often implemented a time-limited goal, typically losing weight to look better, terminating app use upon goal accomplishment. Similarly, Régnier and Chauvel (2018) found MyFitnessPal users, typically aiming for a slimmer physique, used the app for several weeks to months, a few times a year. Lieffers et al. (2018) also reported interviewees’ intermittent use of health-tracking technologies, however some reported persistent use, for up to 4 years, but authors did not report participants’ rationale for long-term use.

Four papers addressed barriers to continued health-tracking technology (Honary et al., 2019; Lieffers et al., 2018; Régnier and Chauvel, 2018; Slazus et al., 2022). Honary et al. (2019) and Slazus et al. (2022) highlighted that participants reported discontinuing app use because logging data was burdensome and time-consuming. Similar views were shared by Lieffers et al.’s (2018) interviewees. In contrast, Régnier and Chauvel (2018) reported that some participants viewed logging food as a way to make time for oneself outside domestic or professional obligations. Other barriers reported included lack of trust in apps and a belief that app use leads to obsessive food behaviours (Slazus et al., 2022).

Overall, the reviewed literature suggests that the most common motivators for initiating health-tracking technology use were managing weight and shape via food-tracking. For those using activity-tracking apps, fitness and sport performance goals were more likely to be motivators. It may be that users of costly activity-tracking devices (e.g. Garmin watches), have the financial resources to not only purchase the device, but also to adopt preventative approaches to health and fitness by consuming a higher-quality diet.

### Process of logging food

The literature highlighted that practicalities of logging food intake appeared to influence users’ eating habits. Hahn et al. (2021b) identified that almost all 18 participants reported increased awareness of their food choices, specifically the types of food they were/were not eating and consequently, they reportedly spent more time thinking about food. Self-monitoring was also associated with increased reflection on eating habits, encouraging ‘improvements’ (Lieffers et al., 2018), as well as users reporting to perceive food items in terms of their calorific content (Didžiokaitė et al., 2018). Interestingly, recording home-cooked meals with multiple ingredients was found to encourage some users to favour ready meals that could be logged quicker (Honary et al., 2019; Lieffers et al., 2018). Lieffers et al.’s (2018) sample discussed challenges associated with quantifying portion sizes, leading to several participants buying weighing scales to increase accuracy. This impact of food logging was echoed by over half of Slazus et al.’s (2022) participants.

It therefore appears that the process of logging food can encourage users to actively engage with their own eating habits, including identifying gaps in dietary intake, at least in the short-term. For some, logging food data was associated with focusing on calorific content and prioritising ease of recording over the nutritional content of food. For others, it seems logging food encourages precision, with users starting to weigh food in order to accurately input data.

### Reification of data

A number of articles hinted at participants’ attitudes towards the data itself. Two studies identified a perspective that data gave an activity its value (Honary et al., 2019; Régnier and Chauvel, 2018), with interviewees stating that when an activity was not recorded, it was as though it never happened. In the same study (Honary et al., 2019), two female interviewees described cheating by not logging ‘bad’ foods, presumably to not tarnish their food intake records. This seems to imply that participants offered more value to their data profiles than their true experiences; the data reified the lived experience or behaviour.

Several papers identified participants who were using more than one type of health-tracking technology concurrently. Hahn et al. (2021b) recruited 20 female participants to use MyFitnessPal for 1 month and found that participants voluntarily began other types of self-monitoring, including exercise and water intake. Two further studies found that participants often used more than one type of health-tracking technology (Hahn et al., 2021c; Sarcona et al., 2017) and Simpson and Mazzeo (2017) found a positive correlation between calorie-tracking and fitness-tracking among 493 undergraduate students. Overall, it appears that a proportion of health-tracking technology users afford high value to the appearance of their data profile, with the data seemingly reifying the sense of achievement, perhaps more so than the action or experience of the activity itself. Moreover, it is possible that initiation of self-monitoring may encourage users to begin to collect data on a wider range of their behaviours.

### Disordered eating and compulsive exercise

Over half of the studies highlighted negative outcomes of health-tracking technology use, including disordered eating and compulsive exercise. Two studies noted that participants followed the calorie limit offered by the technology without question (Didžiokaitė et al., 2018; Lieffers et al., 2018), with only some noting that food-tracking apps often do not account for individual needs. Interviewees also reported to worry about receiving app reminders at inappropriate times or not feeling comfortable entering data around others (Honary et al., 2019; Lieffers et al., 2018). As engagement with apps is largely private, the pursuit of privacy may be a risk factor for intensifying isolating behaviours commonly seen in eating disorder presentations.

The literature identified a range of negative feelings associated with self-monitoring. Following 1 month of MyFitnessPal use, almost half of Hahn et al.’s (2021b) sample reported increased self-consciousness around food choices, body shape and weight or guilt and worry. Similarly, almost half of 95 survey participants indicated they had experienced guilt because of the persuasive nature of apps, social isolation due to personal regimens around diet and fitness goals, and felt controlled by the app (Honary et al., 2019). Males in particular discussed feelings of failure when not achieving their goals. Female participants interviewed by Lieffers et al. (2018) shared that use of health-tracking technologies could be ‘addictive’ and ‘overcome life’ to promote an unhealthy obsession with calories, food, and weight.

In relation to disordered eating behaviours, female and male undergraduate students who reported using weight-related self-monitoring were more likely to report fasting, skipping meals, and excessively exercising (Hahn et al., 2021c). However, males who only used activity-tracking did not have an increased likelihood of disordered eating behaviours. In contrast, an online survey of undergraduate students found that activity-tracking, but not calorie-tracking, uniquely accounted for eating disorder symptomology after adjusting for gender and bingeing and purging behaviour within the past month (Simpson and Mazzeo, 2017). Individuals who used calorie-trackers also manifested higher levels of eating concern and dietary restraint than non-users. Simpson and Mazzeo (2017) concluded that activity-tracking may be a mechanism for promoting exercise for appearance rather than health reasons. Although causality cannot be inferred, evidence suggests that use of food-/calorie-tracking, activity-tracking, or multiple health-tracking technologies may increase the likelihood of development of disordered eating behaviours.

Linardon and Messer (2019) found almost 40% of their male survey participants perceived MyFitnessPal as contributing to eating disorder symptoms. MyFitnessPal users reported significantly higher levels of attitudinal (dichotomous thinking, shape, weight, and eating concerns) and behavioural (binge eating, dietary restraint) eating disorder symptoms than non-users, and scored significantly higher on the Eating Disorder Examination Questionnaire (EDE-Q; Fairburn and Beglin, 1994). Higher scores on the EDE-Q for health-tracking technology users was also observed by Plateau et al. (2018).

Lastly, compulsive exercise was also associated with health-tracking technology use (Gittus et al., 2020; Hahn et al., 2022; Honary et al., 2019; Plateau et al., 2018). Hahn et al. (2022) identified, among women and men, activity-tracking and food-tracking were associated with greater prevalence of disordered weight-control behaviours (e.g. fasting, purging) and disordered muscle-building behaviours (e.g. using steroids). Although activity-tracking and food-tracking were also associated with a higher prevalence of recommended weight-control behaviours (e.g. exercising, changing eating habits), this was only observed amongst those who were also engaging in disordered behaviours. In a survey of undergraduates, Plateau et al. (2018) found that health-tracking technology users reported significantly higher scores on the Compulsive Exercise Test (CET; Taranis et al., 2011), indicating increased compulsive exercise. Additionally, participants using self-monitoring to manage weight and shape reported increased eating and compulsive exercise psychopathology than those using self-monitoring to improve fitness. Similarly, participants in a Fitbit intervention group reported more exercise for appearance-related reasons than control participants, although these effects were nonsignificant (Gittus et al., 2020), and activity goals offered by apps, such as 10,000 steps a day, were reported to overshadow intrinsic motivation for exercise, leading to pursuing activity goals for data purposes rather than for health benefits (Honary et al., 2019).

Overall, health-tracking technologies have been associated with a variety of disordered behaviours. Perceiving the technology as authoritative appears to influence acceptance of pre-set food and activity goals without question and a focus on goal completion as opposed to mindful engagement with eating or exercise to increase wellbeing. Although the reviewed evidence suggests individual motivations for use might impact the likelihood of negative outcomes, findings offer contradictory conclusions. However, the prevalence of negative feelings, disordered eating behaviours, and compulsive exercise appears to be common within the literature.

### Goal achievement and positive self-appraisal

Several studies found that participants reported positive experiences of health-tracking technologies, with their experiences aligning more closely with the developers’ intended aims. Bracken and Waite (2020) found greater MyFitnessPal use was positively associated with self-reported nutrition goal achievement, particularly for individuals with lower self-efficacy for healthy eating, suggesting it was beneficial for individuals who might otherwise feel unlikely to achieve their goals. Likewise, weight management progress and healthy eating was attributed to app use by participants in other studies (Honary et al., 2019; Lieffers et al., 2018; Plateau et al., 2018; Slazus et al., 2022).

Health-tracking technology use was reported as being associated with positive emotions, specifically participants reporting feeling physically and emotionally better on days when they stayed within the daily calorie recommendations (Hahn et al., 2021b). Although some individuals reported that apps prompted obsessive exercise behaviours, they appreciated the push to do exercise even when tired (Honary et al., 2019). Sarcona et al. (2017) found that health-tracking technology use was related to participants feeling better about themselves, feeling like an athlete, and motivating others to participate in exercise and weight loss. App users also reported higher scores on the Eating Behaviour Inventory (O’neil and Rieder, 2005) than non-users, with higher scores indicating positive eating behaviours. Notably, positive emotions elicited by app use appear to be based on compliance with pre-set targets, calling into question the apparent benefits of individual users persistently pursuing such targets.

In two articles, health-tracking technology use had neither positive nor negative impacts on eating behaviours. In their RCT, Gittus et al. (2020) found participants wearing a Fitbit for 10 days did not show any elevation in exercise engagement, motivations for exercise, body satisfaction, or disordered eating symptoms compared to the control group. Hahn et al.’s (2021a) RCT found that MyFitnessPal use for 1 month did not change participants’ likelihood of fasting, compulsive exercising, bingeing, or food restriction. It was also reported that the intervention did not affect secondary mental health outcomes including anxiety, depressive symptoms, body image, or quality of life.

In general, health-tracking technologies can be seen to assist users in achieving health goals, which can be linked to increased positive emotions and self-appraisal. However, in some cases, this positive self-appraisal appears to be associated with (sometimes pre-set) goal achievement, rather than a shift in self-efficacy or wellbeing.

## Discussion

This systematic search and review of research focusing on users’ experiences of health-tracking technologies in relation to their relationship with food and eating identified 15 studies. Overall, the reviewed literature suggests that most common motivators for initiating use of calorie-tracking or activity-tracking technologies are managing weight and shape as a preventative approach to health, a reactive response to weight gain, or possibly intentions to pursue unhealthy weight loss. Due to largely self-report methods, it is unclear whether weight loss resulting from app use moves users towards their healthy weight range or further from it. Better understanding is required regarding who might benefit from health-tracking technology and what assumptions about motivations for use are required to maximise benefits. Such knowledge could guide how health-tracking technologies in health contexts need to be framed for them to be useful and minimise risk of disordered eating habits.

Several reviewed studies highlighted that practical aspects of self-tracking can influence individuals’ eating habits. Logging all food consumed can encourage individuals to pursue accuracy, either through weighing food or choosing readymade meals. Such an approach tends to imply a focus on precise calorific content, highlighted by the app, rather than nutritional content. Similarly, literature suggests a proportion of users afford high value to their data, which could encourage relentless, obsessional pursuit of rigid goals or even additional self-monitoring. The notion that some users ‘cheat’ via selectively recording food intake implies the data morphs in function, from capturing their intake to perhaps representing an idealised or public version of themselves. It is of note that, although the UK’s National Health Service’s Long Term Plan offers the benefits of wearable technologies for managing public health (i.e. weight management), the nature of their use, and the implications of shifting responsibility for monitoring health to individuals rather than medical professionals, needs to be further explored.

Lastly, a considerable proportion of the studies identified a range of concerning experiences associated with health-tracking technology use, at times associated with viewing the technology as an authoritative presence that took precedence over social aspects of eating. Furthermore, as engagement with apps is largely private, this may be a risk factor for intensifying isolating behaviours commonly seen in eating disorder presentations. If disordered eating habits arise following technology use, further exploration of user experiences and reflection on the role of technology, and decisions-making about whether to continue or cease app use, would be desirable in future research, as well as in clinical settings with individuals vulnerable to developing disordered eating or obsessional behaviours.

Contradictory conclusions are offered in the literature regarding the influence of individual motivators and app choice on disordered behaviours, but the prevalence of negative feelings (i.e. guilt, shame, self-criticism), disordered eating, and compensatory behaviours appears to be common amongst users participating in the reviewed studies. However, there does appear to be a group of individuals for whom health-tracking technologies can have a positive influence on health and wellbeing, with users reporting positive experiences of technology use. It may be beneficial to further explore whether certain app functions (e.g. notifications, goal settings, or duration of app use) are associated with differential risk. Findings could inform app development, including offering specific in-app recommendations on how to use the technology in a manner that minimises risk for disordered eating behaviours.

### Limitations

Limitations of the literature included in this review pertain to the research designs employed, study settings, and populations sampled from, for example, reliance of researchers on large samples of university students for studies examining correlations that are therefore unable to provide causal explanations of observed phenomena. Similar research on adults over 30 years of age is scarce, which limits our understanding of the influence of health-tracking technologies on eating habits and attitudes, as well as the development and maintenance of disordered eating presentations, across the lifespan. Moreover, long-term outcomes of health-tracking technology use remain unclear due to very limited follow-up periods reported.

Studies included in this review were predominantly from the Western world, which may overshadow cultural differences in eating behaviours and self-tracking across the globe, as technology is rarely designed in an absence of cultural influences. For example, Lu et al. (2021) found that Chinese food-tracking apps use persuasive design principles to promote thinness, an ideology heavily influenced by cultural norms, whereas Western counterparts were considered to prioritise health rather than centring body image.

Recruitment methods of reviewed studies likely also impacted their sample composition, that is, Sarcona et al. (2017) recruited participants by offering healthy snacks on campus and students motivated by healthy snacks may hold certain views about health and diet. Two studies recruited only females (Hahn et al., 2021a, 2021b) and one recruited only males (Linardon and Messer, 2019). Considering Hahn et al. (2021c) identified gender-specific patterns of health-tracking technology use, further studies focusing on male samples would be valuable, particularly as psychometric properties of some eating scales have not yet been validated in male samples (Linardon and Messer, 2019).

Finally, as the identities of researchers can influence their approach to science (Roberts et al., 2020), the authors wish to disclose the following information about their backgrounds. Two of the authors self-identified as female and one as male, all self-identified as white and, with respect to relevant professional experience, two worked in eating disorders services and one in a diabetes service. A limitation of the review, related to author demographics, is the inclusion of only papers in English and within the specified databases; further research may be available in other languages and databases. It is also important to note that, although the informal quality rating supported narrative development, it did not enable differential weighting of evidence from trials in the synthesis.

### Conclusion

Initiation of self-tracking using a device or app can lead to novel eating habits. Sometimes such novel habits appear to be helpful through promoting self-reflection on food intake and gaps in nutrition, however increased self-awareness may also prompt some individuals to adopt rigid approaches to food intake, including disordered eating behaviours. Introduction of detailed data regarding dietary consumption and activity levels can be associated with an unhelpful focus on the data itself as opposed to mindful engagement with food and enjoyable activity. The precision offered by many apps can also convey an unrealistic approach to human functioning, with the possibility of measuring portions to the gram and exercise represented as exact calorie equivalents. Further research would be welcomed, particularly that focused on long-term outcomes of health-tracking technology use, and cultural and gender differences in the use of such technology, all of which remain unclear due to limitations in the design of studies contributing to the current evidence base.
